# Comparison of Capture Hi-C Analytical Pipelines

**DOI:** 10.3389/fgene.2022.786501

**Published:** 2022-01-28

**Authors:** Dina Aljogol, I. Richard Thompson, Cameron S. Osborne, Borbala Mifsud

**Affiliations:** ^1^ College of Health and Life Sciences, Hamad Bin Khalifa University, Doha, Qatar; ^2^ Qatar Biomedical Research Institute, Hamad Bin Khalifa University, Doha, Qatar; ^3^ Department of Medical and Molecular Genetics, King’s College London, London, United Kingdom; ^4^ William Harvey Research Institute, Queen Mary University of London, London, United Kingdom

**Keywords:** epigenetics, gene regulation, computational pipeline, capture Hi-C, chromatin organization

## Abstract

It is now evident that DNA forms an organized nuclear architecture, which is essential to maintain the structural and functional integrity of the genome. Chromatin organization can be systematically studied due to the recent boom in chromosome conformation capture technologies (e.g., 3C and its successors 4C, 5C and Hi-C), which is accompanied by the development of computational pipelines to identify biologically meaningful chromatin contacts in such data. However, not all tools are applicable to all experimental designs and all structural features. Capture Hi-C (CHi-C) is a method that uses an intermediate hybridization step to target and select predefined regions of interest in a Hi-C library, thereby increasing effective sequencing depth for those regions. It allows researchers to investigate fine chromatin structures at high resolution, for instance promoter-enhancer loops, but it introduces additional biases with the capture step, and therefore requires specialized pipelines. Here, we compare multiple analytical pipelines for CHi-C data analysis. We consider the effect of retaining multi-mapping reads and compare the efficiency of different statistical approaches in both identifying reproducible interactions and determining biologically significant interactions. At restriction fragment level resolution, the number of multi-mapping reads that could be rescued was negligible. The number of identified interactions varied widely, depending on the analytical method, indicating large differences in type I and type II error rates. The optimal pipeline depends on the project-specific tolerance level of false positive and false negative chromatin contacts.

## 1 Introduction

The DNA fiber within the nucleus is assembled into an organized, multi-level architecture. During interphase, chromosomes occupy distinct territories that rarely interact (Cremer and Cremer 2010). Chromatin is further partitioned into hubs of active and inactive compartments, determined by their chromatin accessibility status, gene density and bound proteins (Rao et al., 2014). These compartments are built from smaller topologically associated domains (TADs), which serve as regulatory units, enclosing most chromatin loops within their boundaries (Dixon et al., 2012; Nora et al., 2012). Chromatin loops facilitate the communication of distant genomic regions by bringing them into physical proximity, including enhancers and their target promoters. Substantial evidence supports the importance of this organization in maintaining genome integrity and driving key biological processes, such as transcription (Osborne et al., 2004; Rao et al., 2014; Rhie et al., 2019; Akdemir et al., 2020; Cai et al., 2021). For instance, 3D genomic rearrangements allow genes to alternate between areas of active and repressed chromatin environments to regulate the circadian rhythm (Furlan-Magaril et al., 2021).

The 3D genome architecture can be investigated using either imaging or chromosome conformation capture (3C)-based methods. Imaging techniques are traditionally limited to studying a handful of loci at a time, even though recent developments in the field allow genome-scale studies (Su et al., 2020). 3C-based methods, on the other hand, have been used to study interactions genome-wide for more than a decade. 3C is a proximity ligation-based method, which was developed by Dekker et al. to study ‘one to one’ contacts using PCR amplification for detection (Dekker et al., 2002). Subsequently, several large-scale methods emerged, including the unbiased, genome-wide method, Hi-C, which leverages high-throughput sequencing to quantify all interactions simultaneously (Lieberman-Aiden et al., 2009). While Hi-C can provide information for all contacts, it requires deep sequencing to confidently identify true genomic interactions at higher resolution (Rao et al., 2014). To overcome this limitation and to focus on regulatory loops, library enrichment strategies, such as Capture Hi-C (CHi-C) (Mifsud et al., 2015) and Capture-C (Davies et al., 2016), have been applied. CHi-C uses sequence-specific RNA baits to further select regions of interest from a pool of ligated Hi-C contacts prior to sequencing. It has been widely used to capture promoter interactions with regulatory elements (Furlan-Magaril et al., 2021; Jung et al., 2019) and it has also been employed to assess disrupted genomic interactions of disease risk loci (Baxter et al., 2018; Song et al., 2019).

A typical Hi-C data analysis workflow includes the following steps: quality control and alignment of sequenced reads (Servant et al., 2015; Wingett et al., 2015; Zheng et al., 2019), optional binning of interactions, bias-correction (Imakaev et al., 2012) and performing a statistical test to identify valid (Mifsud et al., 2017) or functional interactions (Heinz et al., 2010; Hwang et al., 2015; Durand et al., 2016; Ron et al., 2017; Wolff et al., 2018; Kaul et al., 2020), which can be interrogated in downstream analyses (Lajoie et al., 2015). For each step, there is a growing selection of tools. While systematic comparisons of Hi-C analytical pipelines exist (Forcato et al., 2017; Pal et al., 2019), there is a lack of similar comparisons for CHi-C data.

Data from CHi-C experiments requires specialized software because CHi-C-specific biases, such as variable capture efficiency, are not accounted for by most Hi-C analysis tools. Furthermore, bait-bait interactions need to be treated separately from bait-other interactions. Ligation fragments that are targeted by baits on both ends have different capture probabilities compared to those targeted only on a single end.

The main decision points for CHi-C data analysis are choosing the method for alignment and filtering of the sequenced read-pairs and choosing the method for identifying interactions of interest. For alignment, most methods will utilize read pairs, where both ends are aligned uniquely to the genome, e.g., HiCUP and HiC-Pro (Servant et al., 2015; Wingett et al., 2015). Zheng et al. proposed an alternative method that rescues those multi-mapping read pairs that can be unambiguously assigned to an interaction, however, the benefit of this method for CHi-C has not been assessed (Zheng et al., 2019). For identification of interactions of interest, there are a number of distinct strategies. GOTHiC aims to identify those interactions that are not experimental artefacts, but represent real contacts in the nucleus. It does not take genomic distance between the interacting fragments into account and it does not infer biologically relevant interactions (Mifsud et al., 2017). Although it was originally developed for Hi-C data, its visibility correction method, which uses all reads mapping to a fragment as the basis of correction, is applicable to bait-other interactions of CHi-C data as well. In combination with a random ligation sample, a modified version of the algorithm can be applied to bait-bait interactions, which uses a mixed additive/multiplicative model for visibility correction (Mifsud et al., 2015). Other methods aim to find functional interactions by assuming that contacts, which occur more often in the nucleus than other contacts spanning similar genomic distances, are biologically relevant. CHiCAGO’s goal is to identify functional interactions by pinpointing those that show higher contact frequencies than would be expected by Brownian motion of the chromatin. It also corrects for visibility of a fragment by separating baits and other ends into groups of fragments with similar coverage (Cairns et al., 2016). CHiCANE calculates the significance of an interaction taking into account both the genomic distance between two bins and the “interactibility” of bait fragments. “Interactibility” is defined as the number of trans reads a bait fragment has (Holgersen et al., 2021). The above mentioned methods use global background measures to identify real or functional interactions, which do not take into account the local chromatin environment of a given bait fragment. In contrast, CHiCMaxima does not take into account the global properties of the CHi-C data set, but treats the contacts of each bait as a virtual 4C instead. It smoothes the read count profile of the bait and uses local maxima to find those fragments that form functional chromatin loops (Zouari et al., 2019). Here, we compare the performance of these various CHi-C data analysis pipelines in detecting reproducible interactions that are of potential biological relevance.

## 2 Materials and Methods

### 2.1 CD34^+^ CHi-C

#### 2.1.1. CD34^+^ Cell Collection, Purification and Fixation

CD34^+^ cells were collected from the femoral heads of healthy donors who underwent total hip replacement surgery (in a consented study approved by the London - Westminster Research Ethics Committee - IRAS#220344). Bone marrow was extracted and irrigated in Iscove Modified Dulbecco Medium/10% Fetal calf serum. CD34^+^ cells were isolated from the cell suspension using a Dynabeads CD34 Positive Isolation Kit (Invitrogen cat# 11301D). PBS-EDTA washed cells were fixed with 2% final concentration of formaldehyde for 10 min at room temperature. After quenching the fixation with 0.125M final concentration of glycine, CD34^+^ cells were purified using CD34^+^ MicroBeads (Miltenyi) according to manufacturer’s instructions. A 1 ml aliquot was used to assess the CD34^+^ purity by FACS and the purity was determined to be above 90%.

#### 2.1.2. Promoter Capture Hi-C

Hi-C library generation was carried out as described previously (Mifsud et al., 2015), with minor modifications. Briefly, after overnight digestion with HindIII at 37°C, DNA ends were labelled with biotin-14–dATP (Life Technologies) using a Klenow end-filling reaction. In nucleus ligation was performed by ligating together biotinylated DNA ends overnight using T4 DNA ligase (Invitrogen). After phenol: chloroform/ethanol purification DNA was quantified using Qubit, with a maximum of 40 μg taken forward. DNA was sheared to a peak concentration of ∼ 400 bp, using the manufacturer’s instructions (Covaris). Sheared DNA was then end-repaired, polyadenylated, and double size selected using AMPure XP beads to isolate DNA ranging from 250 to 550 bp in size. Ligation fragments marked by biotin were immobilized using MyOne Streptavidin C1 DynaBeads (Invitrogen) and ligated to paired-end adaptors (Illumina). Hi-C libraries were then amplified using PE PCR 1.0 and PE PCR 2.0 primers (Illumina) with 6 PCR amplification cycles.

Promoter capture was carried out with SureSelect target enrichment, using a custom-designed biotinylated RNA bait library and custom paired-end blockers according to the manufacturer’s instructions (Agilent Technologies). The 120-mer baits were targeting both ends of HindIII restriction fragments that overlap with Ensembl promoters of protein-coding, noncoding, antisense, snRNA, miRNA and snoRNA transcripts, had a 25–65% GC content, their sequence contained no more than two consecutive Ns and were within 330 bp of the HindIII restriction fragment terminus. After library enrichment, a post-capture PCR amplification step was carried out using PE PCR 1.0 and PE PCR 2.0 primers with 4 PCR amplification cycles. CHi-C libraries were sequenced on the Illumina HiSeq 2000 platform for paired-end sequencing.

### 2.2 Tools and Datasets

Three replicates of GM12878 in solution ligation promoter capture Hi-C and three replicates each of iPSC and iPSC-derived cardiomyocyte in nucleus promoter capture Hi-C data were downloaded from ArrayExpress (E-MTAB-2323 and E-MTAB-6014, respectively) using fasterq-dump v2.9.6. GRCh37 reference genome and chromosome sizes were obtained from the UCSC genome browser.

H3K27ac, H3K4me1 and H3K4me3 peaks and DNase I hypersensitivity sites (DHS; GM12878, H1) from the Roadmap Epigenomics Consortium (2015), heart DHS from the ENCODE project (Consortium 2012) and Nuclease accessible sites (NAS; CD34^+^) from Gargiulo et al. (2009) were downloaded using the AnnotationHub v2.22.1 R BioConductor package for GM12878 (record numbers “AH29709”, “AH29060”, “AH29061”, and “AH30743”, respectively) and CD34^+^ cells (record numbers “AH42424”, “AH42192”, “AH42194”, and “AH5085”, respectively), H1 cells (record numbers “AH29891”, “AH28878”, “AH28880”, and “AH29873”, respectively) and left ventricle/heart (record numbers “AH30592”, “AH29554”, “AH29555”, and “AH25530”, respectively). Significant H3K27ac, H3K4me1, H3K4me3 and DHS peaks were defined as q-value< 0.05.

**FIGURE 1:**
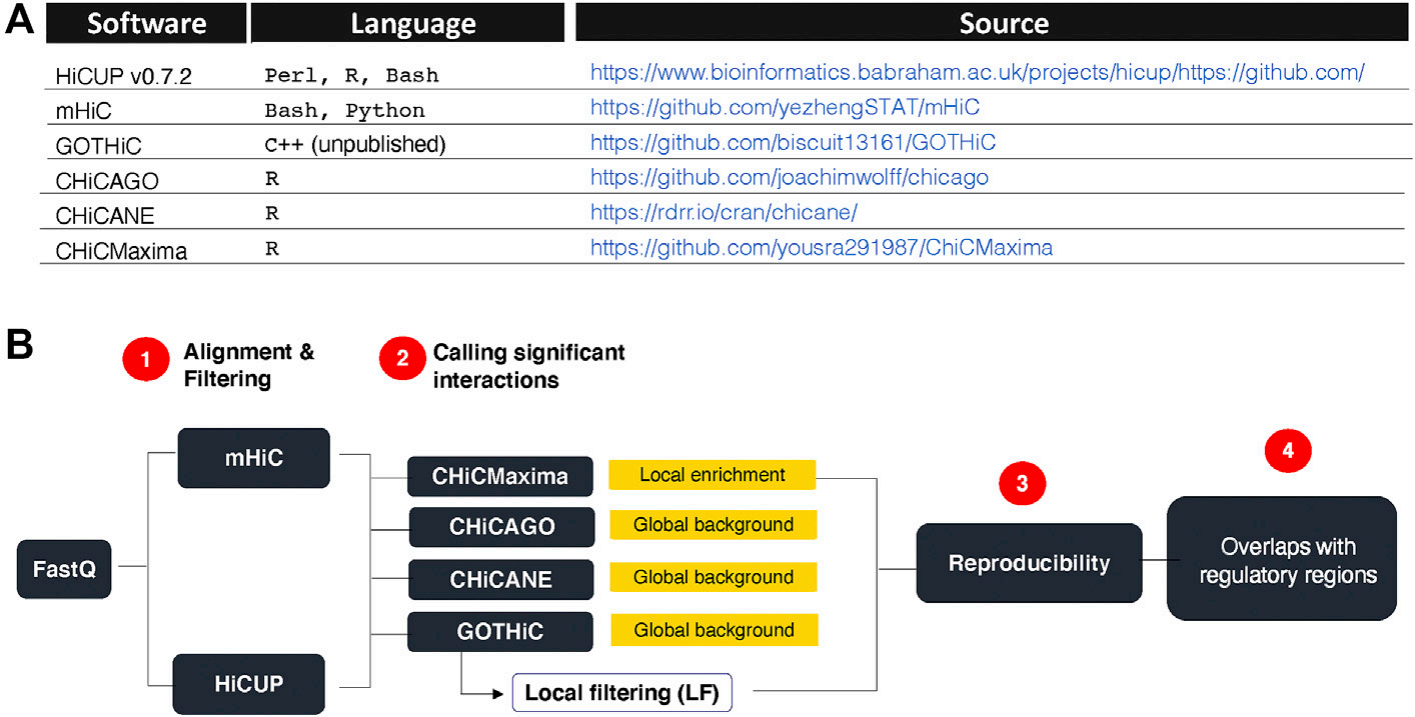
Research summary. **(A)**. CHi-C analytical tools used and their sources. **(B)**. Strategy overview. HiCUP and mHiC were compared for their performance in mapping read pairs and filtering experimental artefacts. GOTHiC, CHiCMaxima, CHiCAGO and CHiCANE were compared for their ability to identify reproducible, biologically relevant interactions. Yellow boxes indicate the type of background model used by each tool. GOTHiC local filtering (LF) is an optional downstream filtering of GOTHiC globally significant interactions based on the local interaction profile of each bait.

HiCUP v0.7.2 (Wingett et al., 2015), mHiC (Zheng et al., 2019), GOTHiC++ (based on (Mifsud et al., 2017), CHiCAGO (Cairns et al., 2016), CHiCANE ((Holgersen et al., 2021) and CHiCMaxima (Zouari et al., 2019) were downloaded from links summarized in Figure 1A.

### 2.3 Read Alignment and Filtering

#### 2.3.1 HiCUP

An *in silico* 1-based HindIII digest profile of the hg19 reference genome was created using hicup_digester. This file represents all possible HindIII fragments in the genome and was used to identify CHi-C artifacts. HiCUP v0.7.2 was used with bowtie2 v2.4.2. (hg19) for the alignment step, and minimum and maximum di-tag ranges were set to 150 and 800 for the filtering step. All other parameters were kept as default. The final BAM output was filtered to include only read pairs where both ends have a mapping quality ≥10.

#### 2.3.2 mHiC

mHiC was applied at four different resolutions: Restriction fragment level (RF), 10 kb, 100 kb and 1 MB with the default BWA aligner (v0.7.17-r1188). The 0-based HindIII digest profile supplied by mHiC was used for mapping the reads to restriction fragments. Parameters were adjusted to be consistent with the parameters used for HiCUP. We used 150 for the minimum and 800 for the maximum di-tag length. The chimeric read length threshold was adjusted to 20. The mapping quality threshold was reduced to 10. The unique and multi-read valid pairs (those that map to unique bins) were concatenated for further processing. The final normalization steps of mHiC were omitted. Valid read pairs were kept from the SAM output of step 2 and the SAM file was converted to BAM using samtools v1.9. for GOTHiC, CHiCAGO and CHiCANE input.

### 2.4 Identifying Significant Interactions

Significant chromatin contacts were identified at fragment resolution using four different software. Three compare the observed read counts for each interaction to a global background and one identifies significant contacts based on the local interaction profile (Figure 1B). Additionally, since the GOTHiC algorithm does not aim to identify functional interactions among those present in the nucleus, we defined bait-specific q-value thresholds for the GOTHiC results. Bait-specific q-value thresholds filter for interactions that are more significant than the majority of contacts a given bait makes. These are likely to represent functional loops.

#### 2.4.1 CHiCAGO

CHiCAGO requires five input files: Rmaps represent all the possible fragments in the genome. Baitmaps represent intervals of fragments that were baited, as well as their bin ID relative to the rmap, and gene names within each captured fragment. The remaining three input files were created using chicagoTools makeDesignFiles.py script with its default settings for HindIII. For MboI-digested fragment we used binsize 1,500, minFragLen 75, maxFragLen 12,000 and maxLBrownEst 97,500. We converted BAM files to chinput format using chicagoTools bam2chicago.sh. Lastly, runChicago.R was executed with the same settings mentioned above. The significance threshold was set to a score ≥5. We also tested ≥10 and ≥15.

#### 2.4.2 CHiCANE

Interactions files were created using prepare. data () with the default parameters and three input files: HiCUP/mHiC BAM files, and the baitmaps and rmaps created previously. We then executed chicane() using the interactions file as input. Significance threshold was set to q-value < 0.05. We also tested <0.01 and <0.001.

#### 2.4.3 GOTHiC**++**

We executed gothic using the BAM files with default settings. Significance threshold was set to q-value < 0.05. We also tested <0.01 and <0.001. GOTHiC identifies interactions that are not due to random ligation events. In order to identify which one of the non-random interactions might be biologically relevant, we defined a per bait q-value threshold based on the slope of the cumulative significance [-log10 (q-value)] curve of the interactions each bait made. Briefly, significance values of all significant interactions of the bait were rounded and for each value we calculated the number of interactions with equal or higher significance. We took the derivative of this cumulative curve to set the threshold for the bait to the significance level, where the absolute slope is above 1.

#### 2.4.4 CHiCMaxima

Interactions input files were created in the format specified in CHiCMaxima. IDs were defined as their bin ID relative to the rmap file. CHiCMaxima was used with default settings with a window size of 20 and 100 for HindIII- and MboI-digested samples, respectively. CHiCMaxima excludes genes with insufficient coverage. Therefore, the output includes only valid interactions.

### 2.5 Downstream Analyses

We assessed the reproducibility of significant interactions two-fold. First, we overlapped the non-bait captured fragments for each bait across all replicates, then we investigated those that overlap with active chromatin. Interactions that were present in at least two replicates were considered reproducible. We also calculated the number of interactions, which pass a joint mean q-value or score threshold in at least two replicates for each tool.

To assess whether significant interactions are of biological relevance we calculated the proportion of identified promoter interacting fragments that harbour active chromatin regions. We overlapped the fragments, or the fragments extended on both sides with either 2.5 kb or 20 kb, with H3K27ac, H3K4me1, H3K4me3 and DNase I hypersensitivity sites using the GenomicRanges v1.42.0 R package.

## 3 Results

### 3.1 The Effect of Multi-Mapping Reads

**FIGURE 2:**
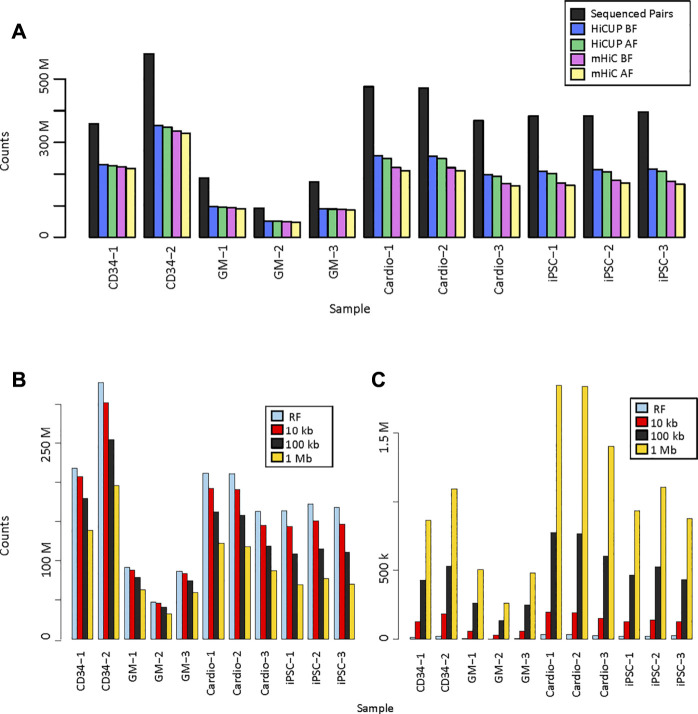
Valid read pairs. **(A)**. Number of identified valid pairs using HiCUP and mHiC. Black bars indicate the total number of raw read pairs. Read pairs were filtered to keep only those with a mapping quality (MAPQ) ≥10. BF: number of mapped read pairs before MAPQ filtering. AF: number of mapped reads after MAPQ filtering. **(B)**. Number of uniquely mapping read pairs using mHiC at different resolutions. **(C)**. Number of rescued multi-mapping read pairs using mHiC at different resolutions. RF: restriction fragment.

We analysed eleven promoter capture Hi-C (PCHi-C) data sets. Three replicates of the GM12878 cell line were in solution-ligation PCHi-C data sets with 93 million to 188 million sequenced read pairs. Two replicates prepared by in nucleus ligation from CD34^+^ hematopoietic stem cells were sequenced more deeply and had 359 million and 579 million read pairs. Three replicates each of iPSC and iPSC-generated cardiomyocyte in nucleus ligation PCHi-C libraries were sequenced at similar depth with 368 million to 475 million reads (Figure 2A, Supplementary Table S1). Raw sequencing reads were mapped and filtered either by HiCUP, which only considers uniquely mapping reads, or by mHiC, which rescues those multi-mapping reads that map to unique restriction fragments or interaction bins, depending on the resolution. HiCUP returned a slightly higher number of valid read-pairs than mHiC, which difference became more prominent in the MboI-digested samples. Each sample had 52–64% valid mapped read-pairs using HiCUP and 45–62% using mHiC. The difference remained the same when only those read-pairs were kept, where both ends had a good mapping quality (MAPQ ≥10) (Figure 2A, Supplementary Table S1.).

Zheng et al. showed that mHiC can rescue up to 20% of reads in Hi-C samples (Zheng et al., 2019), but we did not observe higher valid read counts when mapping these PCHi-C samples. In order to explore whether the lack of improved valid read proportion was due to the high, fragment-level resolution of PCHi-C, we calculated the number of valid unique and rescued multi-mapping reads at fragment level, 10 kb, 100 kb and 1 Mb resolutions (Figures 2B,C, Supplementary Table S2). The number of uniquely mapping reads decreased as the resolution decreased, because a larger proportion of the read pairs fell on the diagonal of the contact matrix, into a single bin, and those read pairs were filtered out. The decrease was more pronounced for deeper sequenced samples and for shorter fragments (Figure 2B). The number of rescued multi-mapping read pairs was negligible at restriction fragment level resolution; at most 32,239 read pairs were rescued in the largest MboI-digested sample. This number did increase with the use of larger bins, however, it did not exceed 1.9M reads at 1 Mb resolution, which was only 0.6–1.6% of the uniquely mapping read pairs in the same samples (Figure 2C).

### 3.2 Reproducibility of Interactions

**FIGURE 3:**
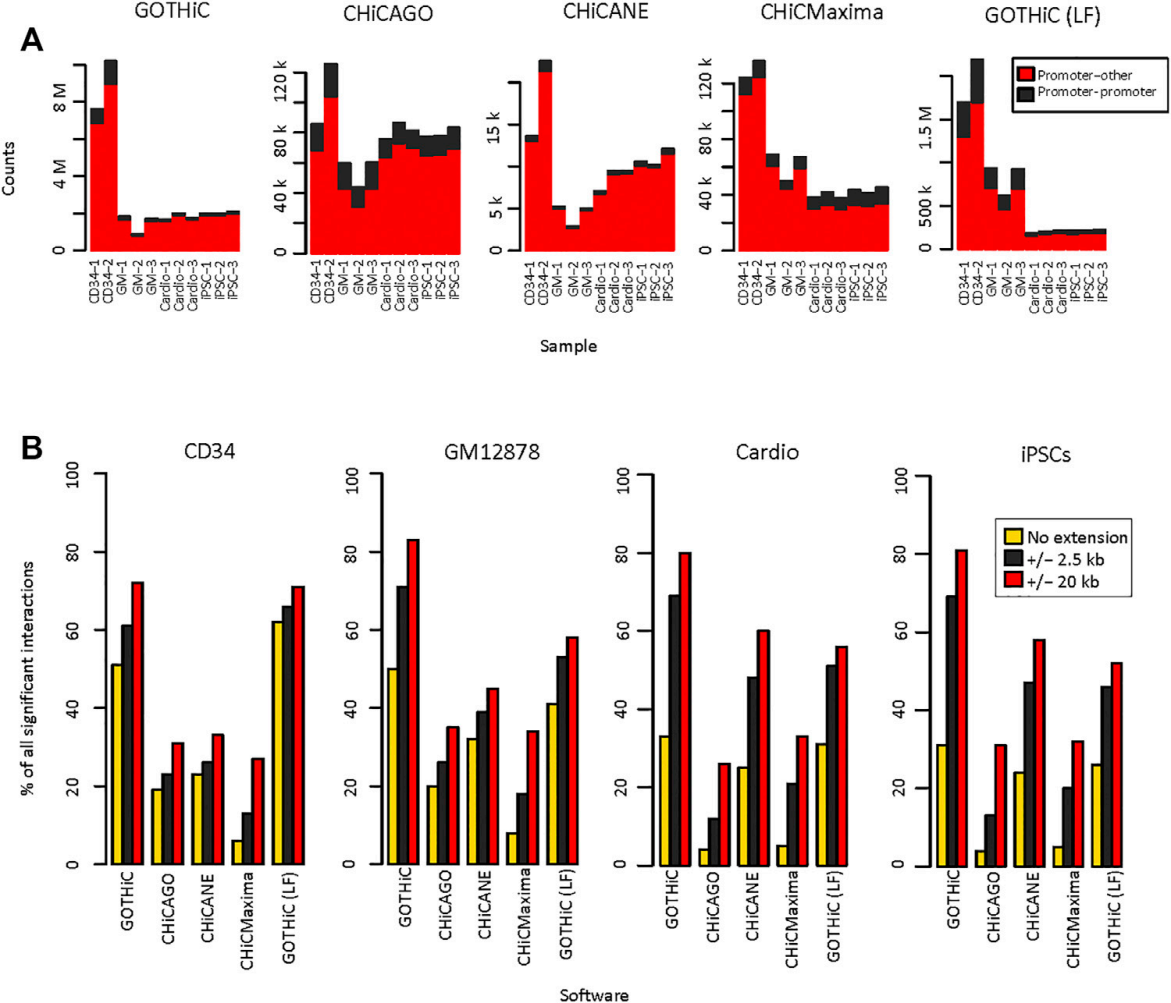
Reproducibility of HiCUP-preprocessed significant interactions. **(A)**. Number of HiCUP-preprocessed significant interactions using GOTHiC (with and without LF), CHiCAGO, CHiCANE and CHiCMaxima. **(B)**. Bar plots represent the percentage of non-baited fragments that overlap in at least two replicates for each bait. Overlaps were studied for exact fragment-level interactions and interactions where non-baited fragment-ends were extended by 2.5 kb or 20 kb.

The numbers of identified significant interactions using HiCUP- or mHiC-aligned and filtered reads were similar. In general, there were up to 10% fewer interactions using mHiC-aligned reads (Figure 3A, Supplementary Figure S1). There was a 2–500-fold difference in the number of interactions identified by GOTHiC and CHiCANE. CHiCMaxima identified slightly more interactions than CHiCAGO and they both returned ∼ 5–20 times as many interactions as CHiCANE (Figure 3A, Supplementary Table S3A). These differences were also apparent in the proportion of baited fragments with at least one identified interaction, which was 99–99.9% and 79–82% with GOTHiC and only 13–89% and 7–13% with CHiCANE for HindIII- and MboI-digested samples, respectively (CHiCAGO: 71–78.9% and 45–52%, CHiCMaxima: 86–99.6% and 32–36%) (Supplementary Table S4). The proportion of bait-bait interactions was highest in CHiCAGO, in the local-filtered GOTHiC and in CHiCMaxima for MboI-digested samples (Figure 3A, Supplementary Figure S1). The number of significant interactions in the 4-cutter-digested samples was equivalent to or lower than the number in the GM12878 datasets by GOTHiC, CHiCMaxima and GOTHiC (LF), despite the deeper sequencing of the iPSC and cardiomyocyte samples. CHiCAGO identified a similar number of significant interactions to those in the larger HindIII samples (Figure 3A, Supplementary Figure S1).

Reproducibility of exact interactions across replicates ranged from 4 to 8% using CHiCMaxima. It was 44–50% for GOTHiC (41–57% after local filtering). CHiCAGO showed 16–20% and CHiCANE interactions showed 22–32% reproducibility (Figure 3B, Supplementary Table S5A). However, when interactions between the exact fragments are not observed, it has been noted that interactions with neighbouring fragments are present in the replicates, therefore we calculated the reproducibility of interactions by extending the non-baited fragments with 2.5 kb or 20 kb on each side. This resulted in a higher proportion of overlapping interactions in all tools, especially for 4-cutter digested samples. The most prominent increase was observed for GOTHiC, the proportion of reproducible interactions increased to 61–71% with the 2.5 kb extension (Supplementary Table S6A) and 70–83%% with the 20 kb extension (Supplementary Table S7A). CHiCMaxima showed the lowest reproducibility after extension as well (Figure 3B). In order to test whether the choice of threshold affected our results, we also filtered at q-values < 0.01 and 0.001 for CHiCANE, GOTHiC and GOTHiC (LF), and at scores ≥ 10 and 15 in CHiCAGO. CHiCMaxima does not have a scoring system and returns only local peaks. Using the second threshold, the number of significant interactions decreased by 11–22% in GOTHiC, 85–96% in CHiCAGO, 41–50% in CHiCANE and 0.3–7% in GOTHiC (LF). Using the third threshold, the number of identified interactions decreased by 28–34% in GOTHiC, 95–99.5% in CHiCAGO, 67–80% in CHiCANE and 0.5–13% in GOTHiC (LF) (Supplementary Tables S3B,C). These had a negligible effect on the reproducibility as the maximum increase was 0–5% in GOTHiC, 1–13% in CHiCAGO, 0–5% in CHiCANE and 0–4% in GOTHiC (LF) at the restriction fragment level (Supplementary Tables S5B,C). The increase was lower when extending for 2.5 kb (Supplementary Tables S6B,C) or 20 kb (Supplementary Tables S7B,C).

The reproducibility of potentially functional interactions, where the non-baited fragments overlapped with DNaseI hypersensitivity sites (DHS), H3K4me1, H3K4me3 or H3K27ac peaks, was higher than it was for all identified interactions using GOTHiC and its local-filtered interaction list but was equal or lower using the other methods (Supplementary Figure S2, Supplementary Tables S8–S10).

All Hi-C-type data, including capture Hi-C, are known to be prone to undersampling, therefore we tested the utility of using a joint mean q-value (GOTHiC, CHiCAGO and CHiCANE) and score (CHiCAGO) threshold for replicates. This resulted in a 0–17% increase in the total number of unique interactions identified across single replicates, the highest being in CHiCAGO. It indicates that, especially in CHiCAGO, many interactions that are significant in one replicate only, are near the threshold in another replicate (Supplementary Table S5D).

### 3.3 Interactions With Potential Biological Function

**FIGURE 4:**
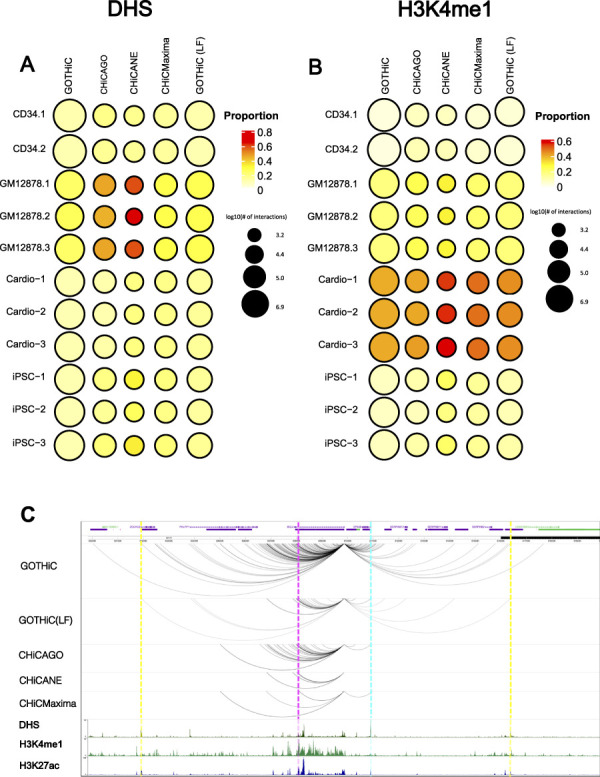
Biologically relevant interactions. Bubble heat maps represent the proportion of interactions that overlap **(A)**. DHS or **(B)**. H3K4me1 peaks. The size of the circle represents the log10 number of promoter-other interactions identified by the different methods. **(C)**. A comparison between *BCL2* promoter interactions in GM12878 cells within ± 1 Mb as reported by each method. Dashed lines represent examples of interactions that are identified in all tools (magenta), all tools except CHiCANE (blue), only in GOTHiC (yellow). ENCODE ChIP-seq profiles for DHS, H3K4me1 and H3K27ac in GM12878 cell lines are shown in the bottom tracks. DHS: DNase I Hypersensitivity sites.

Finally, we assessed whether the identified interactions are of potential biological function, by overlapping the non-baited fragments with DHS (open chromatin), H3K4me1 and H3K27ac peaks (enhancer) and H3K4me3 (active promoter) from the respective cell types. A larger proportion of interactions overlapped with DHS peaks (13–64%) compared to H3K27ac (5–39%), H3K4me1 (5–56%) and H3K4me3 (5–30%) peaks. GOTHiC interactions had the lowest proportion of interactions overlapping with these features. CHiCAGO- and CHiCMaxima-identified interactions had on average a 1.6-fold higher proportion of functional interactions than GOTHiC-identified interactions and 1.2-fold higher than local filtered GOTHiC interactions. In general, CHiCANE showed the highest percentage of interactions overlapping active chromatin (Figures 4A,B). These differences are diminished when the non-baited fragments are extended. 2.5 kb-extended CHiCMaxima interactions have a higher proportion of functional interactions in 4-cutter digested samples than CHiCANE, but lower in 6-cutter digested ones (Supplementary Tables S11–S13).

Interactions made by the baited *BCL2* promoter demonstrate the above observations. Most methods identified a low number of interactions for this promoter, while GOTHiC, even after local filtering, found several interacting fragments. GOTHiC-identified interactions also spanned further than those pinpointed by other methods. CHiCANE interactions were the fewest and shortest. The bottom tracks show DHS, H3K4me1 and H3K27ac profiles in this region. Magenta highlights an interaction that was identified in all methods, blue highlights peaks overlapped with an interaction that was identified by all methods except CHiCANE, while yellow highlights those in GOTHiC-only interacting fragments (Figure 4C).

## 4 Discussion

Recent advances in chromosome conformation capture technologies have enabled us to systematically investigate the spatial arrangement of chromatin within the nucleus. The increasing number of experimental approaches were accompanied by the development of computational pipelines to analyze resulting data and ensure reproducibility of research, but there is no standard method for the analysis of CHi-C data.

Here, we compared two software for the alignment and filtering of reads, HiCUP and mHiC. The former uses uniquely mapping reads only, while the latter keeps those multi-mapping reads that come from a single restriction fragment or genomic bin. At restriction fragment resolution the use of mHiC was not advantageous, in fact HiCUP returned more valid read pairs. This difference might have come from the different aligners used by the two methods, as HiCUP uses Bowtie2, while mHiC uses BWA. For this set of samples, we did not observe substantial benefit from rescuing multi-mapping reads by mHiC at lower resolutions either. However, it might be useful with samples of lower sequencing quality as reads with mismatches are more prone to be misaligned.

Next, we compared methods used for identification of real or functional interactions. In addition to identifying interactions, CHiCAGO and CHiCANE provide R code for functional enrichment and visualization of the data and CHiCMaxima has a graphical interface which facilitates its use for less experienced users. These additional features might also influence the choice of tool. Here we focused on the reproducibility and specificity of regulatory interactions identified.

The most striking difference between these methods was the number of interactions identified. GOTHiC, which identifies those interactions that are not due to spurious ligation of DNA ends, unsurprisingly returns a magnitude higher number of interactions than the other methods, which define interactions as those that are more enriched than their local environment or than other interactions with similar genomic distance. When we aim to find functional interactions and filter GOTHiC results based on the local interaction profiles, there is about 0.1–0.69 of those interactions left, which is still much more than what we found by any other method. It is likely that this method has the highest false positive rate for functional interactions, but examples showed that many regulatory chromatin features are linked to promoters solely by GOTHiC. CHiCANE is the strictest of all four methods tested and a very high proportion of the interacting fragments is overlapping active chromatin, but it is likely to have a very high false negative rate, also indicated by the low proportion of baits with at least a single interaction. CHiCAGO and CHiCMaxima can be a good compromise between false positive and false negative rates, as these identify 4–18 times as many interactions as CHiCANE, and the proportion of those significant interactions that overlap with regulatory features is not much below CHiCANE’s. Extending the interacting fragments with 2.5 kb or 20 kb on each side, increased both reproducibility and the proportion of regulatory interactions. However, the 20 kb-extension reduces the resolution of CHi-C beyond the size of an average regulatory element, which is not recommended for studying promoter-enhancer interactions.

In summary, the choice of method should depend on the tolerable level of false positive and false negative interactions, and this systematic comparison will help researchers identify the method best applicable to their projects.

## Data Availability

The datasets presented in this study can be found in online repositories. The names of the repository/repositories and accession number(s) can be found below: https://www.ebi.ac.uk/arrayexpress/, E-MTAB-10701 https://www.ebi.ac.uk/arrayexpress/, E-MTAB-2323 and https://www.ebi.ac.uk/arrayexpress/ E-MTAB-6014.

## Supplementary Material

Supplementary Figure S1 Number of mHiC-preprocessed significant interactions using GOTHiC (with and without LF), CHiCAGO, CHiCANE and CHiCMaxima.

Supplementary Figure S2 Reproducibility of HiCUP-preprocessed regulatory interactions. Bar plots represent the percentage of **(A)**. DHS, **(B)**. H3K27ac, **(C)**. H3K4me1 or **(D)**. H3K4me3 overlapping non-baited fragments that overlap in at least two replicates for each bait. Overlaps were studied for exact fragment-level interactions and interactions where non-baited fragment-ends were extended by 2.5 kb or 20 kb. DHS: DNase I Hypersensitivity sites.

Supplementary Figure S3 Biologically relevant interactions. Bubble heat maps represent the proportion of interactions that overlap **(A)**. H3K27ac or **(B)**. H3K4me3 peaks. The size of the circle represents the log10 number of promoter-other interactions identified by the different methods.

Supplementary Table S1 Number of mapped valid read pairs using HiCUP and mHiC. Read pairs were filtered to keep only those with a mapping quality (MAPQ) >=10.

Supplementary Table S2 Number of unique and multi-mapping read pairs using mHiC at different resolutions. RF: Restriction fragment.

Supplementary Table S3 Number of significant interactions identified **(A)** at the default thresholds (q<0.05, CHiCAGO score>=5) and at stricter **(B)** q<0.01, score >=10 or **(C)** q<0.001, score >=15 thresholds.

Supplementary Table S4 Number of baits with at least one significant interaction identified. Total number of baits used was 19,022 and 70,545 in HindIII- and MboI-digested samples, respectively.

Supplementary Table S5
**(A-C)**. Percentage of exact non-baited fragments detected at different significance thresholds that overlap in at least two replicates for each bait. **(D)**. Percentage of reproducible interactions, where the average q-value or CHiCAGO score of at least two replicates is below the default threshold.

Supplementary Table S6 Percentage of non-baited fragments detected at different significance thresholds, where both ends have been extended by 2.5 kb, that overlap in at least two replicates for each bait.

Supplementary Table S7 Percentage of non-baited fragments detected at different significance thresholds, where both ends have been extended by 20 kb, that overlap in at least two replicates for each bait.

Supplementary Table S8 Percentage of regulatory interactions that overlap in at least two replicates for each bait. DHS: DNase I Hypersensitivity sites.

Supplementary Table S9 Percentage of regulatory interactions, where both ends have been extended by 2.5 kb that overlap in at least two replicates for each bait. DHS: DNase I Hypersensitivity sites.

Supplementary Table S10 Percentage of regulatory interactions, where both ends have been extended by 20 kb that overlap in at least two replicates for each bait. DHS: DNase I Hypersensitivity sites.

Supplementary Table S11 Proportion of interactions which overlap **(A)**. DHS, **(B)**. H3K27ac, **(C)**. H3K4me1 or **(D)**. H3K4me3 peaks. DHS: DNase I Hypersensitivity sites.

Supplementary Table S12 Proportion of interactions, in which both ends are extended by 2.5 kb that overlap **(A)**. DHS, **(B)**. H3K27ac, **(C)**. H3K4me1 or **(D)**. H3K4me3 peaks. DHS: DNase I Hypersensitivity sites.

Supplementary Table S13 Proportion of interactions, in which both ends are extended by 20 kb that overlap **(A)**. DHS, **(B)**. H3K27ac, **(C)**. H3K4me1 or **(D)**. H3K4me3 peaks. DHS: DNase I Hypersensitivity sites.
