# Supplementary figures and images for: Comparison of Capture Hi-C Analytical Pipelines
Source: Front Genet. 2022 Jan 28;13:786501. doi: 10.3389/fgene.2022.786501 (PMC8859814; doi:10.3389/fgene.2022.786501)

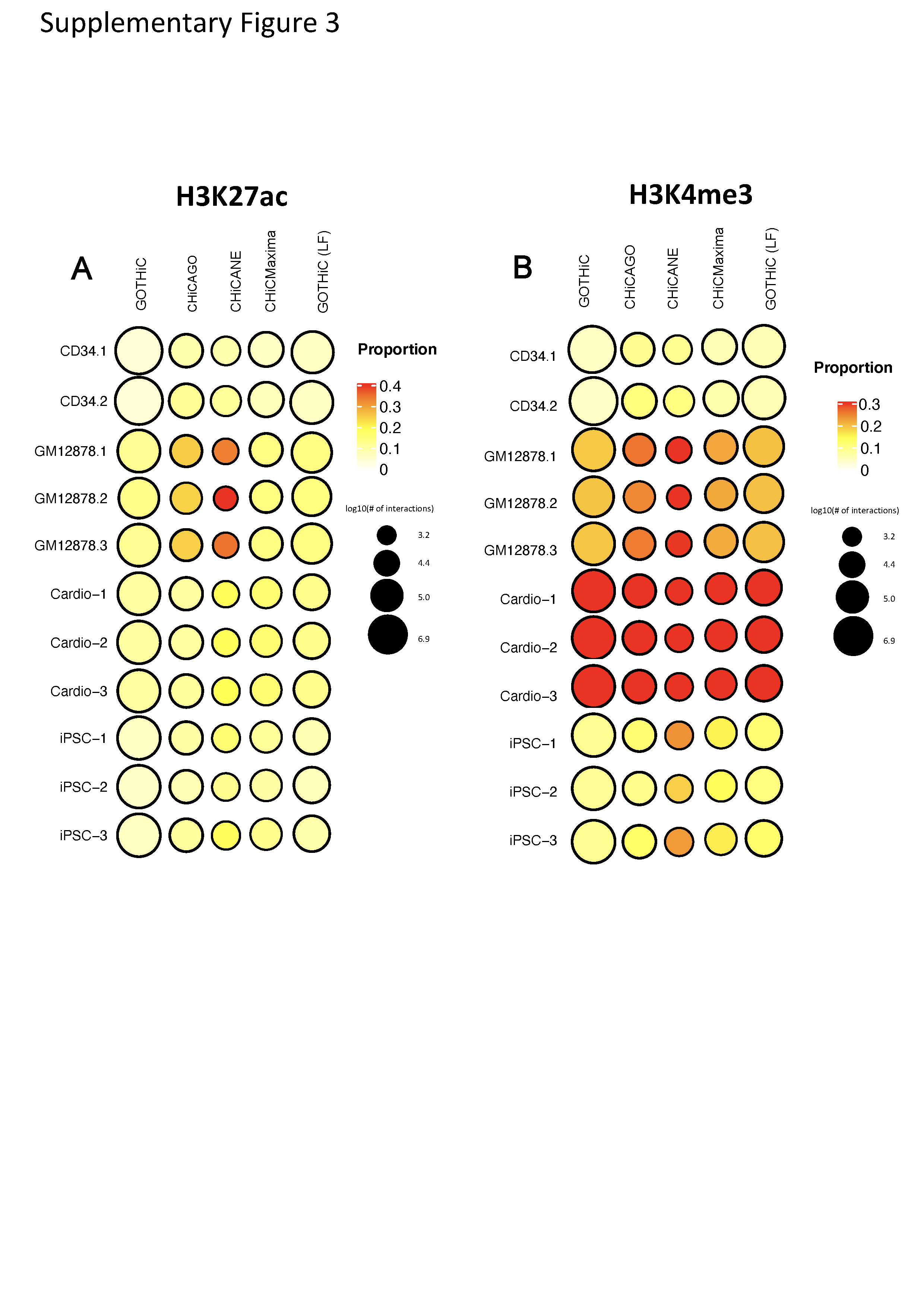

Supplement: Supplementary file 1 [file Image3.TIFF]

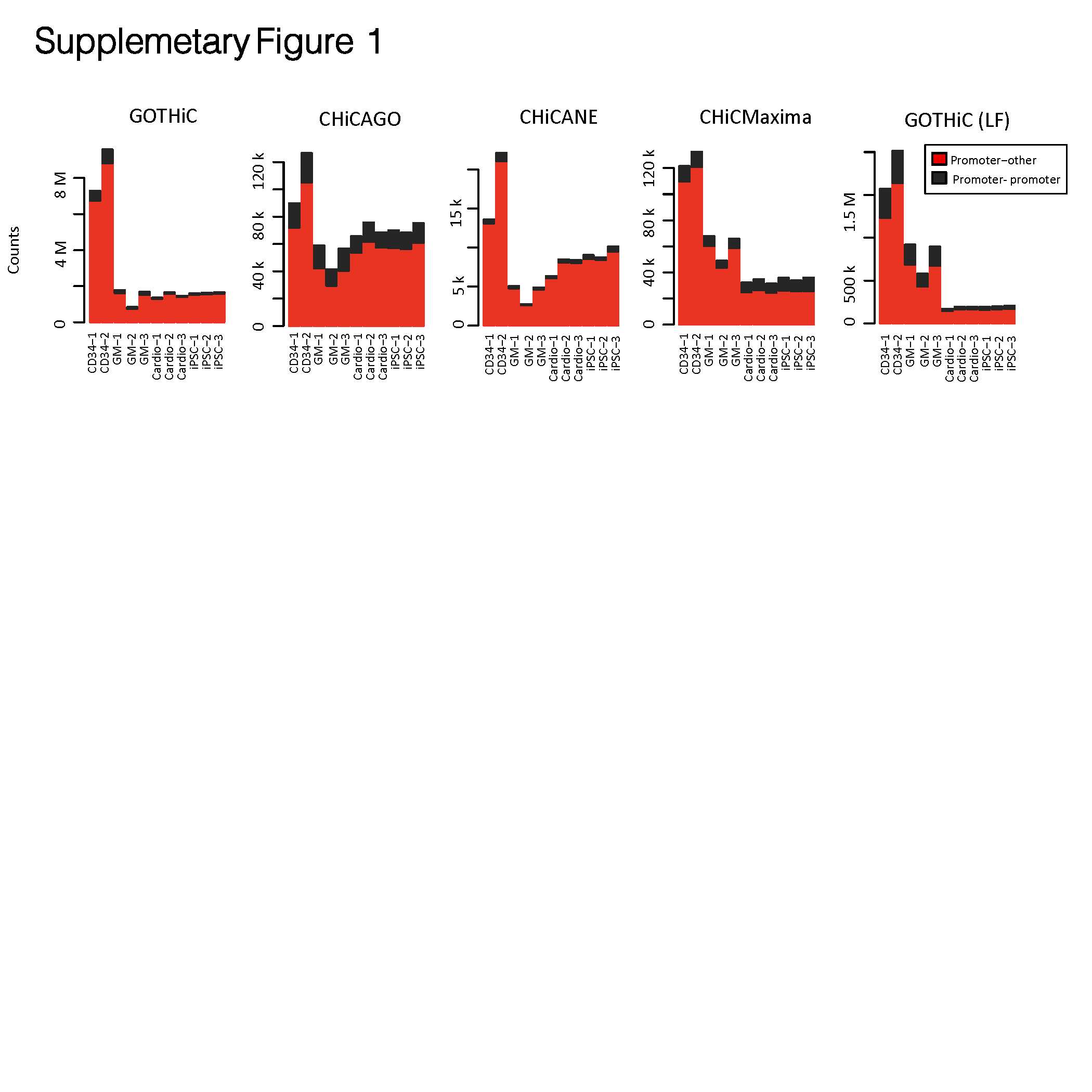

Supplement: Supplementary file 2 [file Image1.TIFF]

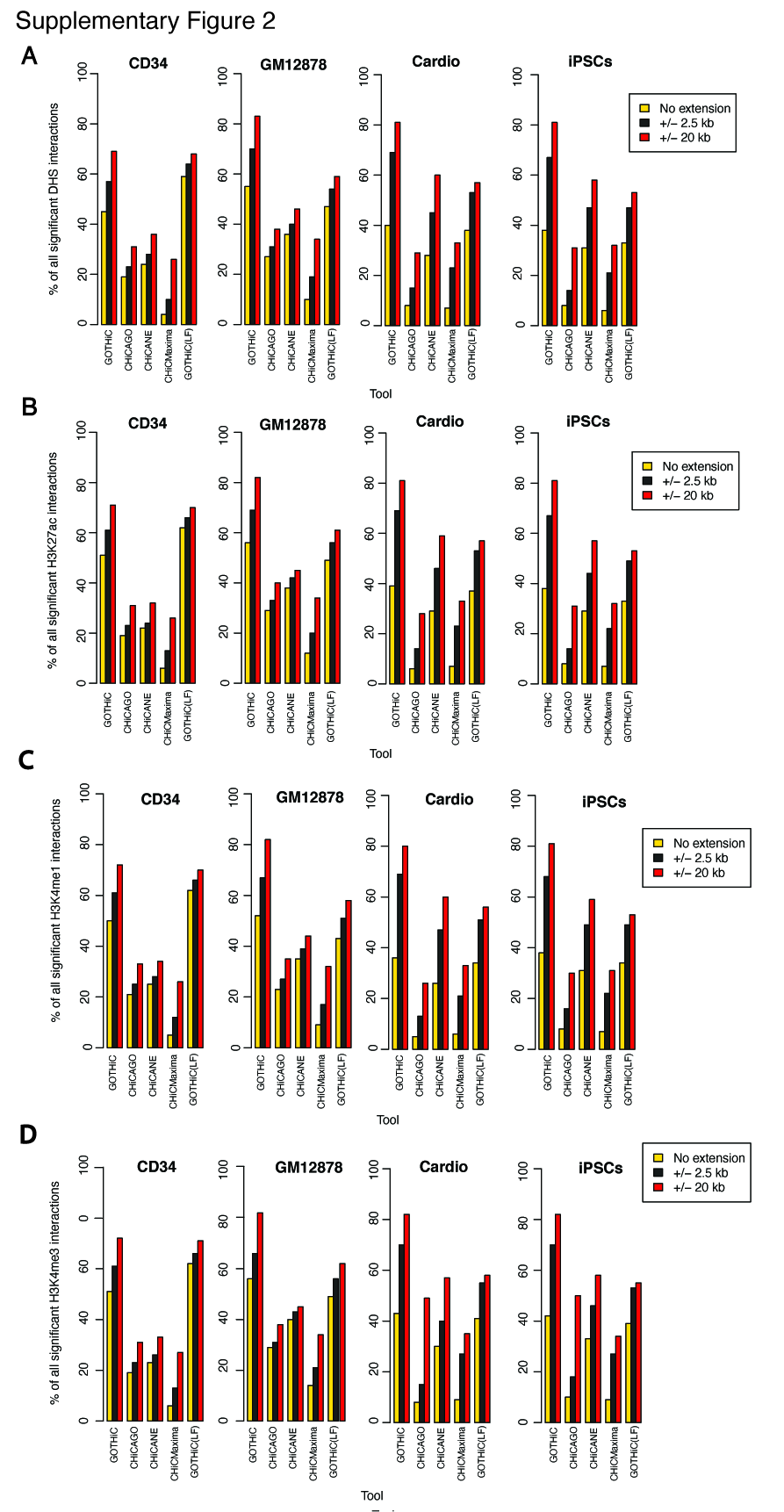

Supplement: Supplementary file 3 [file Image2.TIF]
